# Macrophages Homing to Metastatic Lymph Nodes Can Be Monitored with Ultrasensitive Ferromagnetic Iron-Oxide Nanocubes and a 1.5T Clinical MR Scanner

**DOI:** 10.1371/journal.pone.0029575

**Published:** 2012-01-10

**Authors:** Hye Rim Cho, Seung Hong Choi, Nohyun Lee, Taeghwan Hyeon, Hyeonjin Kim, Woo Kyung Moon

**Affiliations:** 1 Department of Radiology, Seoul National University College of Medicine, Seoul, Korea; 2 Department of Radiation Applied Life Science, Seoul National University College of Medicine, Seoul, Korea; 3 National Creative Research Initiative Center for Oxide Nanocrystalline Materials and School of Chemical and Biological Engineering, Seoul National University, Seoul, Korea; Institute of Hepatology London, United Kingdom

## Abstract

**Background:**

Due to the ability of macrophages to specifically home to tumors, their potential use as a delivery vehicle for cancer therapeutics has been suggested. Tracking the delivery and engraftment of macrophages into human tumors with a 1.5T clinical MR scanner requires the development of sensitive contrast agents for cell labeling. Therefore, this study aimed to determine whether intravenously injected macrophages could target a primary tumor as well as metastatic LNs, and whether these cells could be detected *in vivo* by MRI.

**Methodology:**

Peritoneal macrophages were obtained from BALB/c nude mice. The viability, phagocytotic capacity and migratory activity of the macrophages were assessed. MR imaging was performed using a clinical 1.5 T MR scanner and we estimated the T2* of the labeled macrophages. Metastatic lymph nodes were produced in BALB/c nude mice. We administrated 2×10^6^ macrophages labeled with 50 µg Fe/mL FIONs intravenously into the mice. In the 3D T2* GRE MR images obtained one day after the injection of the labeled macrophages or FION solution, the percentages of pixels in the tumors or LNs below the minimum normalized SI (signal intensity) threshold were summated and reported as the black pixel count (%) for the FION hypointensity. Tumors in the main tumor model as well as the brachial, axillary and inguinal lymph nodes in the metastatic LN models were removed and stained. For all statistical analyses, single-group data were assessed using t test or the Mann-Whitney test. Repeated measurements analysis of variance (ANOVA) with Tukey–Kramer post hoc comparisons were performed for multiple comparisons.

**Conclusions:**

The FION-labeled macrophages, which could be non-invasively monitored using a 1.5 T clinical MR scanner, targeted both the main tumors and LN metastases. Overall, the results of this study suggest that the use of macrophages may have many future applications in the clinic for vectorizing therapeutic agents toward main tumors as well as LN metastases.

## Introduction

Many primary malignancies spread through the lymphatic system. Tumor cells passing through, or residing in, lymph nodes (LNs) can serve as a reservoir of cells that can lead to lethal distant metastases. The detection of metastases in the sentinel LNs and other LNs within the regional bed provides clinically important information for tumor staging, the choice of treatment, and the prediction of patient outcomes [1], [2]. Sensitive and specific noninvasive imaging techniques to visualize LN metastasis *in vivo* are critical for gaining information about tumor progression [3], [4].

Of the various *in vivo* imaging techniques, magnetic resonance imaging (MRI), with its high resolution, exquisite soft-tissue contrast and ability to produce images of entire organs/organisms without the use of ionizing radiation, [4] is the most common method to evaluate a tumor's size, location, and metastatic burden. MRI is especially useful to study cancer dynamics in deep tissues, which makes it readily translatable to clinical applications. MRI has recently emerged as a powerful tool for *in vivo* cell tracking [5], [6] and the simultaneous development of new intracellular contrast agents has allowed the *in vivo* detection of very small cellular populations [7], [8].

The first description of the presence of leukocytes within human tumors, which was thought to reflect the onset of cancer at sites of previous chronic inflammation, was presented by Virchow in 1863 [9]. It has now been established that the majority of malignant tumors contain numerous macrophages as a major component of the host leukocytic infiltrate [10]. These macrophages are referred to as tumor-associated macrophages and although most are derived from peripheral blood monocytes that are recruited into the tumor mass from the circulation [11], [12], there is also evidence of local proliferation of macrophages within the tumor tissue [13], [14]. Chemokines (chemotactic cytokines) provide the directional stimulus for the movement of leukocytes during development, hemostasis, and inflammation, and are believed to be important for the recruitment of monocytes into tumors. Some studies have also shown the tumor-targeting potential of macrophages in animal models [15], [16].

This study aimed to determine whether intravenously injected syngeneic macrophages could target a primary tumor as well as metastatic LNs in a mouse model, and whether these cells could be detected *in vivo* by MRI. We utilized macrophages that had been labeled with ferromagnetic iron-oxide nanocubes (FIONs) to facilitate detection by MRI and histology. After an *in vitro* assessment of the efficiency and innocuity of the labeling procedure, we used *in vivo* MRI in combination with histology to demonstrate and monitor the capacity of metastatic cancer cells in the LNs to attract intravenously administrated macrophages.

## Results

### Identification of macrophages within the intraperitoneal cells by FACS

**Figure 1:**
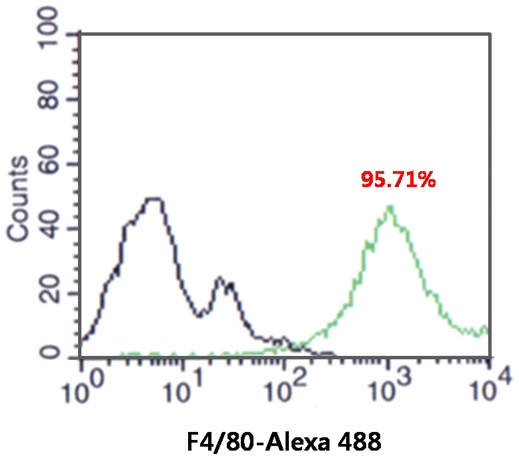
Identification of macrophages in the intraperitoneal cells by FACS. Over 95% of the intraperitoneal cells were F4/80 positive (Black: no staining, Green: F4/80 staining).

Over 95% of the intraperitoneal cells were F4/80 positive (Figure 1).

### Determination of intracellular FION uptake

**Figure 2:**
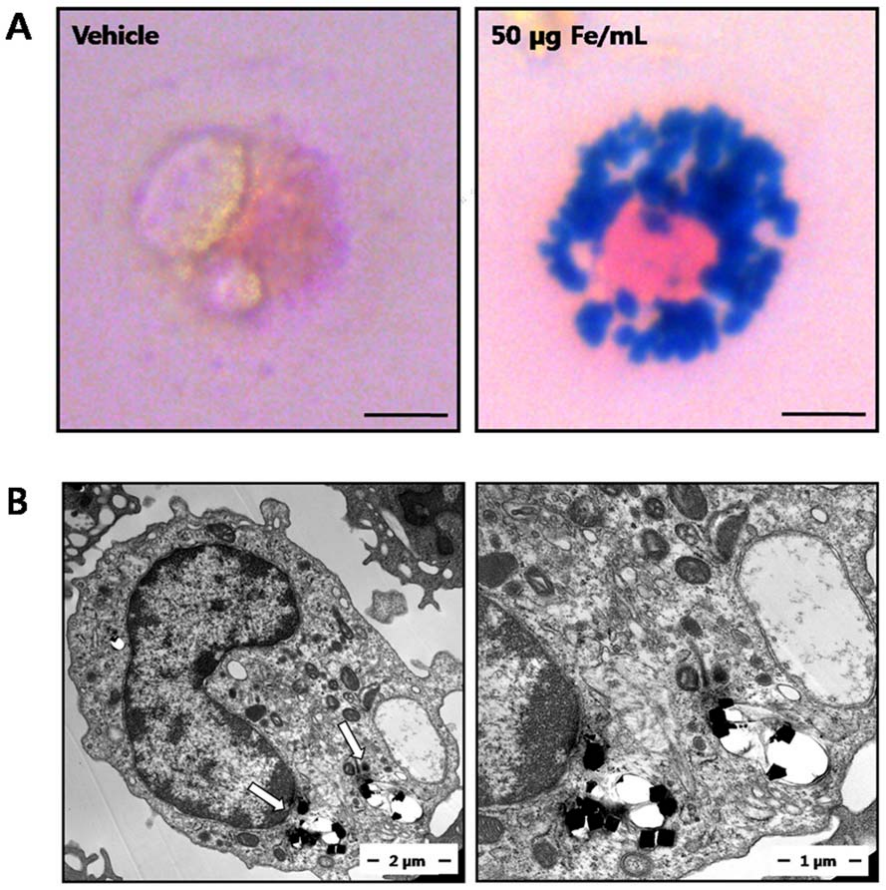
The identification of the intracellular distribution of FIONs in macrophages. (A) Microscopic view (400×) of macrophages treated with FIONs (50 µg Fe/mL). The ingested FIONs were visualized by Prussian blue staining (left: phase, right: Prussian blue). (B) The location of the FIONs was further confirmed by electron microscopy (left: 15000×, right: 30000×), which revealed that the ingested FIONs were located in cytoplasmic organelles (arrows).

After FION labeling using at a concentration of 50 µg Fe/mL for 2 h, Prussian blue staining of the labeled macrophages revealed an abundant uptake of the FIONs into the cytoplasm (Figure 2A). TEM of the FION-labeled macrophages revealed FIONs in the cytoplasmic organelles (Figure 2B).

### The viability, phagocytosis, and migration of FION-labeled macrophages

**Figure 3:**
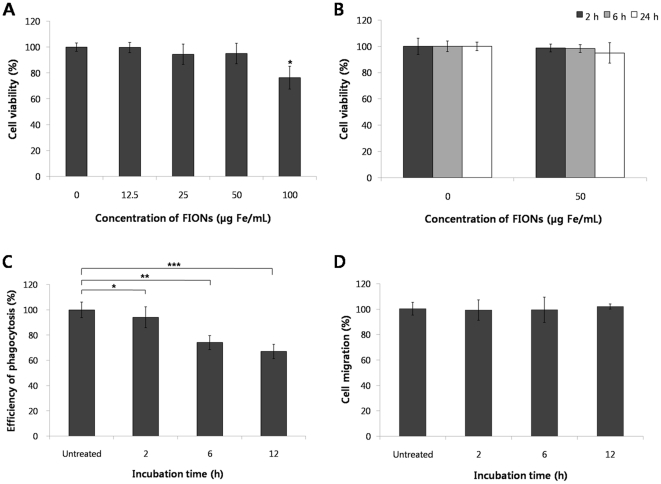
The physiological functions of macrophages after FIONs labeling. (A) Cell viability test of the macrophages after exposure to varying concentrations of the FIONs for 2 h. The cell viability at the concentration of 100 µg Fe/mL FION concentration was significantly lower than the other concentrations tested (* *P*<0.001). (B) No significant differences in the cell viability were observed after treatment with FIONs (50 µg Fe/mL) for varying times (*P* = 0.5393). (C) The efficiency of phagocytosis decreased as the incubation time of the macrophages with the FIONs increased. Macrophages were incubated with FIONs (50 µg Fe/mL) at 37°C for the indicated times, and the efficiency of phagocytosis was determined. The incubation times of 6 and 12 h showed significant differences when compared to the untreated group (* *P*>0.05, ** *P*<0.05, *** *P*<0.01). (D) Macrophages were incubated with FIONs (50 µg Fe/mL) at 37°C for the indicated times, and then a macrophage migration assay was performed. No statistically significant differences were evident between the untreated and the FION treated groups (50 µg Fe/mL). All experiments were analyzed using the ANOVA test and the Tukey-Kramer multiple comparison test.

In terms of cell viability, the MTT assays indicated no statistically significant difference between the macrophages labeled with FIONs at concentrations of 12.5–50 µg Fe/mL and the unlabeled cells. However, the macrophages exposed to the 100 µg Fe/mL FION concentration manifested significantly reduced viability compared with the control cells (Figure 3A). When the macrophages were treated with 50 µg Fe/mL FIONs for up to 24 h, we did not detect any differences in the cell viability (Figure 3B).

The macrophages treated with 50 µg Fe/mL FIONs exhibited reduced phagocytotic efficiency as the incubation time increased from 0 to 12 h. However, no significant difference was detected between the macrophages treated with FIONs for 2 h and the unlabeled cells (Figure 3C).

Additionally, no significant differences in the migratory ability were evident between the unlabeled and FION-labeled macrophages after 2, 6, or 12 h treatments (50 µg Fe/mL FION) (Figure 3D).

### *In vitro* MR imaging

**Figure 4:**
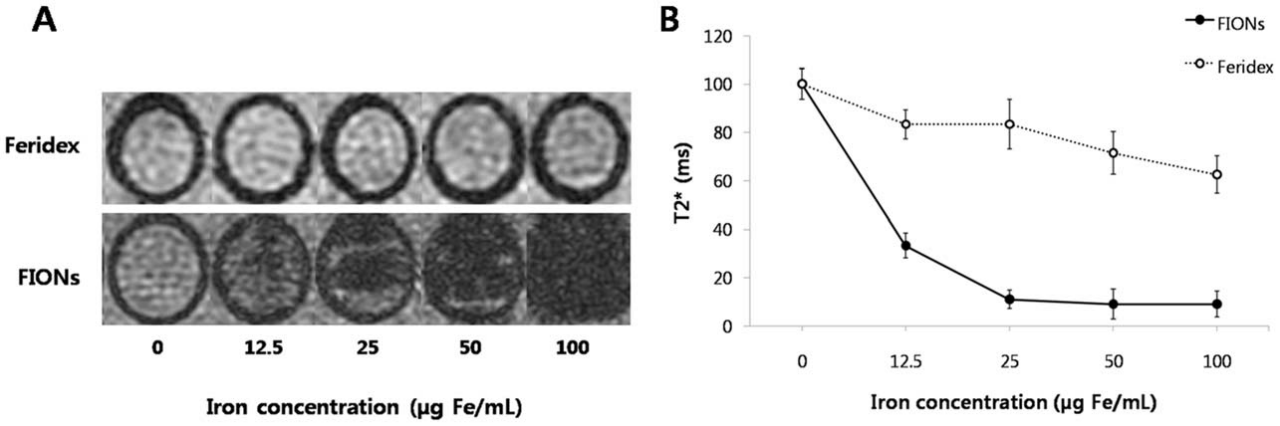
*In vitro* T2* measurement of macrophages labeled with iron oxide. (A) MR phantom of the labeled macrophages was constructed in 1% agar/PBS. T2* MR images showed that the SI significantly decreased when the macrophages were labeled with FIONs. However, the SI of Feridex labeled macrophages was poorly differentiated. (B) The T2* of the macrophages was found to decrease from 100±6.40 ms in the absence of FIONs. The T2* of the FION-labeled macrophages was decreased when the incubation concentration reached 50 µg Fe/mL (9.09±6.02 ms), however, the T2* of the macrophages labeled with 100 (µg Fe/mL) Feridex (62.50±7.69 ms) was higher than that of the macrophages labeled 12.5 µg Fe/mL FIONs (33.33±6.02 ms).

The T2* of the macrophages decreased from 100±6.40 ms in the absence of FIONs to 9.09±6.20 ms in the presence of 50 Fe µg/mL FIONs in the culture medium. This T2* was significantly lower than the T2* (71.43±8.84 ms) of the macrophages labeled with Feridex (Figure 4A). Increasing the iron concentration to 100 Fe µg/mL FIONs did not further reduce the T2* (Figure 4B).

Based on our *in vitro* results, a concentration of 50 µg Fe/mL FIONs and a 2-h incubation time was implemented for the FION-labeling of macrophages for the *in vivo* experiments. These conditions did not affect the physiologic activity of the macrophages.

### *In vivo* MR imaging and histological analysis

#### The main tumor model

**Figure 5:**
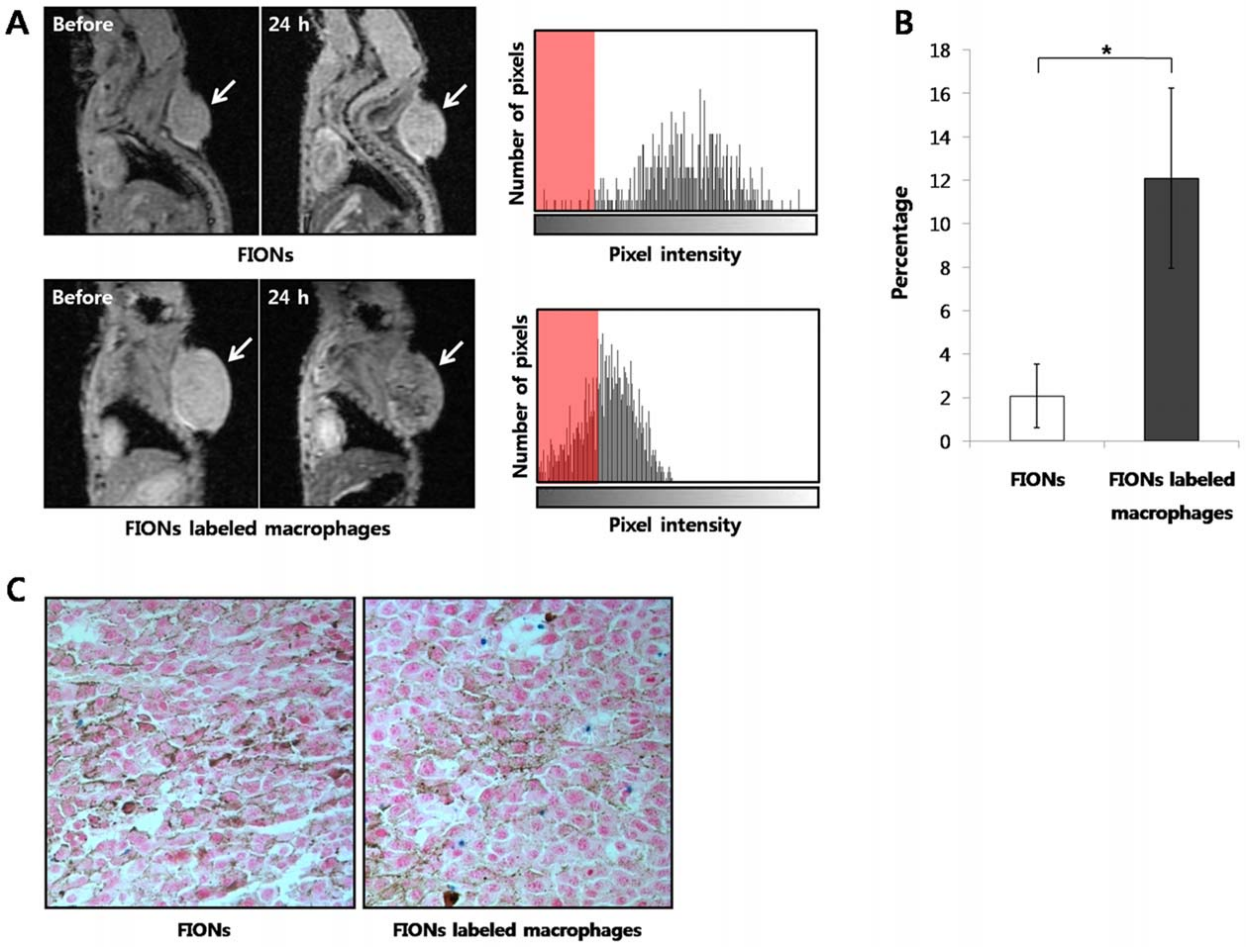
Sagittal T2* GRE MR images of the melanoma tumor model. (A) Sagittal T2* GRE MR images were taken before and 24 h after the intravenous administration of a FION solution or the FION-labeled macrophages. The hypointensities from the FION-labeled macrophages were detected within the tumor on the day after injection. Thus, the hypointensity means the FIONs-labeled macrophages had been recruited within the tumors. Histograms show the primary tumor pixel distributions and those pixels decreasing below the threshold value (Red). Notably, very few pixels in the tumor after the intravenous injection of the FION solution fell below the threshold. (B) The percentages of hypointense pixels within the tumors of the mice treated with the FION-labeled macrophages and the FION solution alone were 12.093±4.139 (%) and 2.074±1.461 (%), respectively (* *P* = 0.0268). (C) Prussian blue staining of the main tumors revealed intracellular FIONs within the macrophages (Blue).

In T2* GRE MR images of the mice with main tumors, the hypointensities from the FION-labeled macrophages were detected within the tumors (n = 6) on the day after the intravenous administration and were found to be, significantly higher than the hypointensities within the main tumors of the mice (n = 6) that received only a FION solution (*P* = 0.00268) (Figure 5A). The percentages of hypointense pixels within the tumors of the mice treated with the FION-labeled macrophages or only a FION solution were 12.093±4.139 and 2.074±1.461, respectively (Figure 5B). Moreover, a histological examination of the main tumors using Prussian blue staining revealed intracellular FIONs within the macrophages (Figure 5C), which was more prominent from the tumors of the mice injected with the FION-labeled macrophages than the FION solution alone.

#### The metastatic lymph node model

A total of 12 brachial, 12 axillary, and 12 inguinal LNs were isolated from the six mice used for the metastatic LN model, and these LNs were evaluated by MRI and histological analysis. In all brachial and axillary LNs, metastases were detected, but metastatic foci were not found in any of the inguinal LNs. The mean sizes of the metastatic melanomas in the brachial and axillary LNs were 8.3 mm (range: 5.2–10.3 mm) and 3.4 mm (range: 1.1–5.4 mm), respectively.

**Figure 6:**
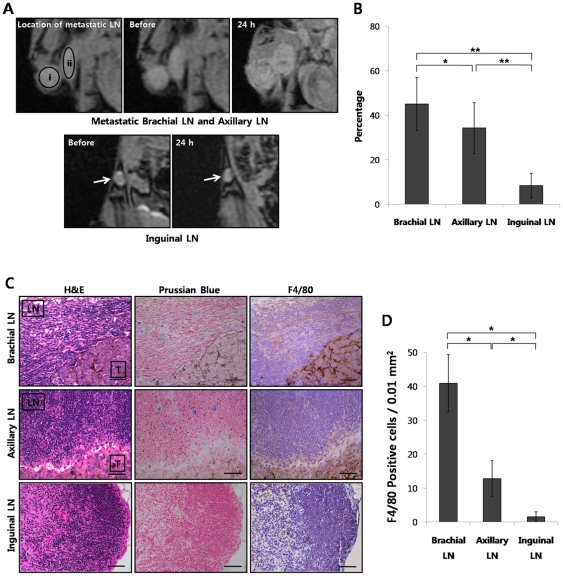
The recruitment of FION-labeled macrophages into metastatic lymph nodes. (A) i and ii represent metastatic brachial and axillary LNs, respectively. Hypointense pixels increased in both the brachial and axillary LNs 24 h after the intravenous injection of FION-labeled macrophages. Non-metastatic inguinal LNs showed fewer hypointensities than the brachial and axillary LNs. (B) The percentages of hypointense pixels within the metastatic brachial and axillary LNs as well as the non-metastatic inguinal LNs were 45.064±11.932 (%), 34.242±11.456 (%) and 8.413±5.449 (%), respectively (* *P*<0.05, ** *P*<0.001). (C) Prussian blue staining demonstrated an abundant presence of FION-loaded cells within the metastatic LNs (Blue). The FION-labeled macrophages in the LNs stained positively with the F4/80 antibody (Brown). LN = lymph node, T = metastatic tumor in the lymph node. (D) The mean numbers of F4/80 positive cells were 40.9±8.4, 12.8±5.3, and 1.6±2.4 cells/ 0.01 mm^2^ in the brachial, axillary and inguinal LNs, respectively, which resulted in statistical significance (* *P*<0.001).

The T2* GRE MR images showed that the hypointensities of the FION-labeled macrophages were detected within the metastatic brachial and axillary LNs on the day after the intravenous administration of the macrophages. In contrast, fewer hypointensities were detected in the non-metastatic inguinal LNs revealed than in the brachial and axillary LNs (Figure 6A). The percentages of hypointense pixels within the metastatic brachial and axillary LNs as well as, the non-metastatic inguinal LNs were 45.064±11.932, 34.242±11.456 and 8.413±5.449, respectively (*P*<0.05). In addition, we could clearly differentiate metastatic LNs from normal LNs using a threshold of 20% hypointense pixels; all LNs exhibiting over 20% of hypointense pixels contained metastases (Figure 6B). Although the FION-labeled cells were present in both the metastatic melanomas and normal LN tissues, Prussian blue staining demonstrated an abundant presence of FION-loaded cells within the metastatic LNs (Figure 6C). The FION-labeled macrophages in the LNs also were also positively stained with the F4/80 antibody (Figure 6C). The mean numbers of F4/80 positive cells in the brachial, axillary and inguinal LNs were 40.9±8.4, 12.8±5.3, and 1.6±2.4 cells/0.01 mm^2^, respectively, and exhibited statistically significant differences (*P*<0.001) (Figure 6D).

## Discussion

Herein, we have demonstrated the feasibility of using FION-labeled macrophages to target main tumors and melanoma LN metastases in mice. Although previous investigations have used macrophages or stem cells labeled with iron oxide particles to target tumors [16], [17], this study is the first to report the use of macrophages labeled with iron-particles to target LN metastases. Additionally, in this study, we have demonstrated that the homing of iron-labeled macrophages can be detected and visualized *in vivo* using a clinical 1.5T MR scanner.

Macrophages have enormous potential as vehicles for directed therapeutic delivery. The mechanism responsible for the homing of activated macrophages to tumors likely involves chemokine ligands and receptors similar to the recruitment of other leukocytes to areas of inflammation. The most important of these chemokines produced by human tumors appear to be monocyte chemotactic protein-1 (MCP-1), macrophage colony stimulating factor (M-CSF or CSF-1), and vascular endothelial growth factor (VEGF) [18]. Hypoxia is also known to recruit of macrophages to the tumor regions [19]. Valable et al [15] visualized macrophages labeled with micrometer-sized iron-oxide particles using MRI and demonstrated that the labeled cells, accumulated in the brain tumors in rats. However, to our knowledge, the present study is the first to assess the applicability of a systemic delivery of iron-particle-labeled macrophages to metastatic tumors in the LNs.

As a non-invasive imaging technique that uses non-ionizing radiation, MRI and the general use of iron particles may have important future roles in human applications. In the case of cellular MR imaging, the labeling method used in this study has several advantages. First, the macrophages could be easily labeled with the FIONs, within a two-hour time periods. Although previous studies have reported that an overnight incubation was required to label the macrophages with Feridex [20], [21], [22], the intracellular iron concentration in the macrophages labeled with FIONs for 2 h was higher than the concentration found after a 24 h Feridex labeling (Figure S2). In theory, the short labeling time would improve the viability and functionality of the transplanted macrophages. Second, the FIONs did not affect the viability or functions, including phagocytosis and migration, of the labeled macrophages. Third, we could visualize the injected FION-labeled macrophages using a clinical 1.5 T MR scanner due to the large magnetization and resulting high relaxivity of the FIONs [23], [24]. Furthermore, the feasibility of using FION particles for tracking a variety of transplanted cells *in vivo* after labeling is known [24]. Until now, however, the use of direct intravenous FION injections has been limited *in vivo* because of the tendency of FIONs to aggregate. For clinical applications, the ability to track the homing of macrophages to primary tumors and LN metastases using a simple non-invasive clinical scanner would be greatly beneficial.

Currently, key challenges for anti-tumoral therapy include maintaining an elevated concentration of therapeutic agents at the tumor site and preventing the spread of these agents into the surrounding healthy tissue [25], [26]. Cell-based targeting strategies for the delivery of therapeutic agents have great potential for overcoming these challenges. Using genetic engineering, cellular therapy may facilitate the sustained production of a desired molecule. For example, genetically modified mesenchymal or neural stem cells overexpressing interleukin-2 [27] or interleukin-12 [28] may be used to inhibit tumor growth. One of the body's responses to the presence of a malignant neoplasm is the recruitment of peripheral blood monocytes to the tumor by a chemoattractive gradient into the tumor. Once the monocytes cross the endothelial basement membrane, they differentiate into macrophages [16]. Macrophages are important components of the innate immune response against tumors and are attracted by locally secreted chemokines as described above [29], [30]. Thus, we believe the approach described here could be utilized for the delivery of anti-tumor agents towards primary tumors as well as LN metastasis.

Other studies have suggested that some of the MRI signal may be the result of either the release of free iron, or the uptake of iron by endogenous macrophages after the death of labeled cells [31], [32]. In our experiments, we did not detect MRI signal with the use of free FIONs, suggesting that the iron is cleared under such circumstances. Thus, the MRI signal is generated exclusively by viable, labeled cells.

In the present study, murine B16F10 melanoma cells were chosen because of their high potential to metastasize to regional LNs [33], [34]. Although the murine models employed in this study have implicated macrophages as promising agents for cancer targeting, some uncertainties still remain. Further study of the homing ability of macrophages to different tumor entities and their hematogenous or lymphatic metastases would be of great benefit for clinical applications.

Bone marrow-derived macrophages, resident or induced peritoneal macrophages, or macrophages from the spleen, liver, and lung can be isolated from mice. The phenotypic and functional data obtained from studies using macrophages isolated from these various sites have indicated that all macrophages are not equal with respect to cytokine production, migratory capacity, and the ability to ingest and kill pathogens [35]. In the present study, we used peritoneal macrophages activated by treatment with thioglycollate broth for the following reasons. First, peritoneal lavage is a common method for obtaining relatively large numbers of fully differentiated macrophages from mice, and approximately 10^7^ macrophages can be recovered per mouse after treatment with thioglycollate broth [36]. Second, the main purpose of the present study was to investigate whether the macrophages labeled with FION can be used to target tumors *in vivo*, so we did not include experiments to determine which type of macrophage is appropriate for targeting tumors. Thus, we believe that further study using macrophages isolated from various sites is warranted for future clinical applications.

In this study, metastatic LN mouse model was obtained by injecting melanoma cells near the brachial LNs, which induced metastasis in brachial and axillary LNs. This method is limited by the difficulty of simulating the metastatic spread condition because of the proximity of the injection site to the LNs. However, we believe that this method has strengths in regard to the study of sentinel LNs and the high yield of metastatic LNs.

In conclusion, this study has demonstrated that macrophages can be simply and efficiently labeled with FIONs. The labeled macrophages, which could be monitored non-invasively using a 1.5 T clinical MR scanner, targeted both main tumors and LN metastases. Overall, the results of this study suggest that the use of macrophages may have many future applications in the clinic for vectorizing therapeutic agents towards main tumors as well as LN metastasis.

## Materials and Methods

These experiments were approved by the animal care committee at Seoul National University Hospital.

### The isolation of peritoneal macrophages

Activated peritoneal macrophages were obtained from six-week-old male BALB/c nude mice. For the murine MR imaging experiments, activated peritoneal macrophages were obtained from syngeneic mice 5 days after the intraperitoneal injection of 2 mL of a 3% aged Brewer thioglycollate solution (Sigma) using peritoneal washings. The peritoneal washings were centrifuged at 1500 g for 10 min and then resuspended in 1 mL of red blood cell lysis buffer (Sigma) for 7 min. Cells were then centrifuged again and resuspended in RPMI-1640 medium (WelGENE) containing 10% fetal bovine serum (WelGENE) and 1% a penicillin-streptomycin mixture (Gibco). The cell suspensions were then plated in tissue culture flasks and allowed to adhere to it [21].

### Identification of peritoneal macrophages by FACS

To identify the macrophages within the intraperitoneal cells, we used a FACSCalibur flow cytometer (BD Biosciences) equipped with a 530-nm filter (bandwidth±15 nm) and a 585-nm filter (bandwidth±21 nm) and Cell-Quest software (BD Bioscience), and the analysis was performed using an F4/80 antibody (Abcam). After removing the red blood cells as described above, 1×10^4^ cells were fixed by 4% paraformaldehyde for 30 min at room temperature. The cells were washed with PBS and stained with primary F4/80 antibody for 1 h. Control cells were not stained with antibody. After 1 h, the cells were washed several times and analyzed by fluorescence after staining with Alexa Flour 488-conjugated secondary antibody.

### Contrast agents for macrophage labeling

For labeling macrophages, FIONs were synthesized and used according to the previously reported methods [23], [24]. Two milliliters of the synthesized FIONs in chloroform were mixed with 1 mL of chloroform containing 10 mg of PEG-phospholipid and 1 mg of 1, 2-distearoyl-*sn*-glycero-3-phosphoethanolamine-*N*-[amino(polyethyleneglycol)-2000] (NH_2_-PEG-phospholipid, Avanti Polar Lipids, Inc.) and dispersed in carbonate buffer (pH 9.0). The size of the PEG-phospholipid encapsulated FIONs was 57.8±9.9 nm. The magnetization of the FIONs at 300 K was measured to be 132.1 emu per gram of Fe. The r2 relaxivity of the FIONs dispersed in a 1% agarose solution was 324 mM^−1^ s^−1^ at 1.5 T, which was higher than had been reported for Feridex® (Advanced Magnetics, Cambridge, Mass) and MPIOs (Bangs Laboratory®, Fisher, IN, USA) dispersed in a 1% agarose solution (133 and 169 mM^−1^ s^−1^ at 1.5 T, respectively) [23], [24].

### The labeling of macrophages and the measurement of iron content

The macrophages were magnetically labeled with FIONs or Feridex, and the intracellular concentration of iron was measured (Figures S1, S2, and Files S1, S2).

### Transmission electron microscopy (TEM)

The labeled macrophages were washed with PBS and then fixed with 2.5% glutaraldehyde in 0.1 M PBS (pH 7.4) for 2 h. Subsequently, the macrophages were treated with 2% osmium tetroxide in 0.1 mM cacodylate buffer for 2 h. The macrophages were then dehydrated using a graded ethanol series (from 50 to 100% ethanol) in propylene oxide (EM Sciences, Fort Washington, PA). The samples were then embedded in pure Epon resin (EM Sciences) for 3 days at 60°C. Next, ultrathin sections were prepared using glass knives and a Diatome diamond knife (Reichert-Jung, Vienna, Austria) on an RMC MTXL ultramicrotome (Tucson, AZ). The prepared sections were then stained with lead citrate and uranyl acetate (both from EM Sciences) and visualized by TEM (JEM-1400).

### Cell viability assay

To determine cell viabilities, macrophages were initially seeded in 96-well tissue culture plates at a density of 1×10^5^ cells per well. The macrophages were washed three times using PBS after exposure to FIONs at concentrations of 12.5, 25, 50, or100 µg Fe/mL for 2 h, or FIONs at a concentration of 50 µg Fe/mL for 2, 6 or 24 h. The viabilities of the macrophages were assessed using a standard 3-,5-diphenyltetrazolium bromide (MTT) assay. The optical densities were read at 540 nm.

### Phagocytosis assay

The bacterial phagocytotic capacity of the macrophages was assayed using a commercially available kit (Vybrant phagocytosis assay kit; Molecular Probes). Macrophages were incubated with or without FIONs dissolved in RPMI-1640 at a 50 µg Fe/mL concentration for varying time (2, 6, or 12 h) at a cell density of 1×10^5^ cells per well in 96-well plates. The media was then removed, fluorescein-labeled *Escherichia coli* were added, and the procedure was performed according the manufacturer's instructions.

### Migration assay

Macrophage migration assays were performed in 96-well chambers (CytoSelect CBA-105; Cell Biolabs) according to the manufacturer's instructions. Briefly, 1×10^5^ macrophages that had been treated with 50 µg Fe/mL FIONs for 2, 6 or 12 h were suspended in the upper chamber, and the media from a 48 h culture of B16F10 cells was placed in the lower compartment of the chamber. After 24 h, the migrated cells were detected with CyQuant GR Dye (Molecular Probes).

### *In vitro* MR imaging of phantom

MR phantom of the labeled macrophages for stable and homogeneous MRI measurement was constructed in 1% agar/PBS. Briefly, peritoneal macrophages were incubated with FIONs or Feridex at concentrations of 12.5, 25, 50, or 100 µg Fe/mL for 2 h in standard tissue culture incubators with 5% CO_2_. After the incubation, 1×10^3^ cells were washed, harvested, transferred to 0.2 mL thin wall strip tubes (Axygen) that had been, coated with 50 µL molten 1% agar/PBS on the bottom and centrifuged (1000 rpm, 5 min). The supernatants were removed, and 50 µL of molten 1% agar/PBS was added and given 30 min at room temperature to settle. All agar/PBS solutions were sterilized through 0.45 µm pore size filter paper (Whatman). MR imaging was performed using a clinical 1.5 T MR scanner (Signa Excite, GE healthcare) with a wrist coil. For the estimation of T2*, we used a gradient echo (GE) pulse sequence with the following imaging parameters: TR/TE = 800/4.9, 13.6, 22.3, or 57 ms; flip angle = 20°, FOV = 50×50 mm, matrix = 256×256, slice thickness = 2.1 mm, and the number of excitation = 2. Regions of interest (ROIs) were defined in the representative slices acquired at the shortest TE, and this data was used to generate the T2* maps of the ROIs using a pixel-by-pixel analysis across the four-point MR images in MATLAB™ (MathWorks Inc., Natick, USA) assuming single exponential decay (i.e., SI = SI_0_×e^−TE/T2*^, where SI represents signal intensity and SI_0_ represents proton density).

### Animal models

B16F10 cells were obtained from the American Type Culture Collection (ATCC, Rockville, MD) and maintained in DMEM with 10% fetal bovine serum (FBS) at 37°C.

#### The main tumor model

B16F10 melanoma cells were prepared in 100 µL serum- free DMEM and then subcutaneously transplanted into the shoulders of 6-week old BALB/c nude mice (n = 12; 2×10^6^ cells/100 µL medium/each mouse). *In vivo* MR imaging of the tumors was performed 2 weeks after the cell implantation.

#### The metastatic lymph node model

Metastatic lymph nodes were produced in 6-week old BALB/c nude mice (n = 6) according to the following step: A. a superficial skin incision, approximately 1 cm in length, was made in the bilateral upper brachial area of anesthetized mice. B. the skin was inverted to expose the brachial lymph nodes. C. a 5 µL volume of 2×10^6^ B16F10 cells was then slowly injected in the fatty area adjacent to the bilateral brachial area using a Hamilton syringe and an investigational microneedle (Hamilton Syringe), and D. the dermal incision was closed with a 5-0 Prolene purse string (Harrell Medical). *In vivo* MR imaging of the metastatic lymph nodes was performed 1 week after the cell implantation.

### *In vivo* MR imaging

*In vivo* MR imaging was performed using a clinical 1.5 T MR scanner (Signa Excite, GE Healthcare) with a wrist coil. The imaging protocol consisted of a sagittal and coronal 3D T2* GRE sequence with the following imaging parameters: TR/TE = 58/12, flip angle = 10°, FOV = 80×80 mm, matrix = 256×192, slice thickness = 0.7 mm, and the number of excitations = 6. Mice were anesthetized by the intraperitoneal injection of a solution containing zolazepam (5 mg/kg, Zoletil®, Virbac) and xylazine (10 mg/kg, Rompun®, Bayer-Schering Pharma). We obtained MR images before and one day after the intravenous administration of the labeled macrophages or a FION solution.

#### Main tumor model

Mice were intravenously injected with 2×10^6^ macrophages that had been labeled with 50 µg Fe/mL FIONs for 2 h (n = 6) or 2×10^6^ macrophages that had been labeled with a FION solution of identical Fe amount (9.1 µg FIONs) (n = 6).

#### Metastatic lymph node model

We administered 2×10^6^ macrophages that had been labeled with 50 µg Fe/mL FIONs for 2 h intravenously into the mice (n = 6).

### *In vivo* MR imaging analysis

The MR data were digitally transferred from a PACS workstation to a personal computer and processed with ImageJ (available at http://rsb.info.nih.gov/ij/ ) and software developed in house using Microsoft Visual C++. One author performed all of the image processing, region-of-interest (ROI) drawing, and data analyses.

ROIs that contained the entire tumor or lymph node (brachial, axillary and inguinal LNs were included) were drawn in each section of the T2* GRE images. Using the software developed in-house, the data acquired from each slice were summated to derive the pixel-by-pixel SI values for the entire tumor. Previous studies [37], [38] have shown that muscle tissue remains unchanged by the contrast agent; therefore, the pixel-by-pixel SI values were then normalized to the muscle to cancel the SI fluctuations related to variations in the technical parameters between the MRI sequences obtained both pre- and post-injection of the labeled macrophages or the FION solution. The SI of muscle was measured within a single ROI measuring 3–5 mm^2^ placed in the shoulder muscle. Finally, SI histograms for the tumors and LNs were plotted using a bin size of 1% with normalized SI (nSI) value (i.e., normalized SI value (%) = [SI of tumors or lymph nodes]/[SI of muscles]×100) on the x-axis, and the percentage of the total lesion volume was expressed on the y axis by dividing the frequency of each bin by the total percentages of pixels analyzed.

A baseline pixel histogram using the nSI of a tumor or LN was created from MR images obtained before the injection of the labeled macrophages or FION solution to establish the minimum nSI in the absence of any FIONs. On the MR images obtained one day after the injection of the labeled macrophages or FION solution, the percentages of pixels in the tumors or LNs below the minimum nSI threshold was summated and reported as the black pixel count (%) for the FION hypointensity.

### Histological analysis

Tumors from the main tumor models (n = 12) as well as brachial, axillary and inguinal lymph nodes from the metastatic LN models (n = 6) were removed and fixed in 10% buffered formalin. Paraffin-embedded tumors and lymph nodes were sectioned into 4-µm thick sections. Staining methods included hematoxylin and eosin (H&E) to visualize the tumor and lymph node morphology, and Prussian blue and immunohistochemical staining. To detect the presence of FIONs in the tissues, prepared paraffin sections were dewaxed, hydrated, and treated with 0.01% protease XXIV (Sigma) in PBS for 20 min at 37°C. Sections were then incubated with a 1∶1 (vol/vol) mixture of 1% potassium ferrous cyanide (kaliumhexacyanoferrat [II]) and 5% HCl for 1 h. Slides were then rinsed in distilled water and counterstained with Nuclear Fast Red for 10 min. The presence of iron oxides was qualitatively assessed with a microscope.

For the detection of mature macrophages in LNs, immunohistochemical staining was performed using the following steps were performed using the F4/80 antibody (Abcam). First, endogenous peroxidase and protein were blocked with a solution of 0.3% H_2_O_2_ and goat serum (Dako) to prevent nonspecific antibody binding. After 30 min of blocking, the tissues were incubated with the primary F4/80 antibody for 1 h, and after several brief washes, a HRP-conjugated goat anti-rat secondary antibody (Santacruz) was applied for 2 h. After additional washings, the macrophages were evaluated by staining with the peroxidase DAB substrate (Dako). The F4/80-positive cells in each LN were counted in a square unit of surface with an area of 0.01 mm^2^. The mean numbers of F4/80 positive cells/surface unit were calculated from 50 measurements in each LN from all of the mice with metastatic LNs.

### Statistical analysis

For all statistical analyses, a two-tailed P value of less than 0.05 was considered to be statistically significant. Statistical analyses were performed using commercially available software (MedCalc, version 11.1.1.0, MedCalc Software, Mariakerke, Belgium). Single-group data were assessed using Student's t test or the Mann-Whitney test. Repeated measurements analysis of variance (ANOVA) with Tukey–Kramer post hoc comparisons were performed for multiple comparisons.

## Supporting Information

Figure S1 **The Prussian blue staining of macrophages after incubation with various concentrations of iron oxide for different times.** With increases in the concentration of iron oxide (from 12.5 to 100 µg Fe/mL) and exposure time (from 2 to 12 h), the macrophages phagocytosed more particles. When compared with Feridex-labeling, the FION-labeled macrophages showed a higher intracellular uptake of iron after a 2 h-incubation period.
(TIF)

Figure S2 **The intracellular iron content (pg Fe/cell) after incubation with increasing iron oxide concentrations (µg Fe/mL).** The iron concentration in the macrophages correlated to the FION incubation concentration. Up to 4.539 (pg) of FIONs could be ingested by one macrophage when the FION incubation concentration reached 50 (µg Fe/mL) for 2 h. The amount of iron was 2.214 (pg) when the FIONs concentration was 25 (µg Fe/mL) for 2 h (•). However, when the macrophages were incubated with Feridex in concentration 100 (µg Fe/mL) for 24 h, the amount of iron ingested was 1.284 (pg/cell) (○ with the dotted line), which was significantly lower (* *P*<0.001) than the iron content of the macrophages treated with different concentration of FIONs (from 12.5–50 µg Fe/mL) for 2 h.
(TIF)

File S1 **Supporting information.**
(DOC)

File S2 **Supporting information.**
(DOC)
