# Supplementary figures and images for: Macrophages Homing to Metastatic Lymph Nodes Can Be Monitored with Ultrasensitive Ferromagnetic Iron-Oxide Nanocubes and a 1.5T Clinical MR Scanner
Source: PLoS One. 2012 Jan 10;7(1):e29575. doi: 10.1371/journal.pone.0029575 (PMC3254614; doi:10.1371/journal.pone.0029575)

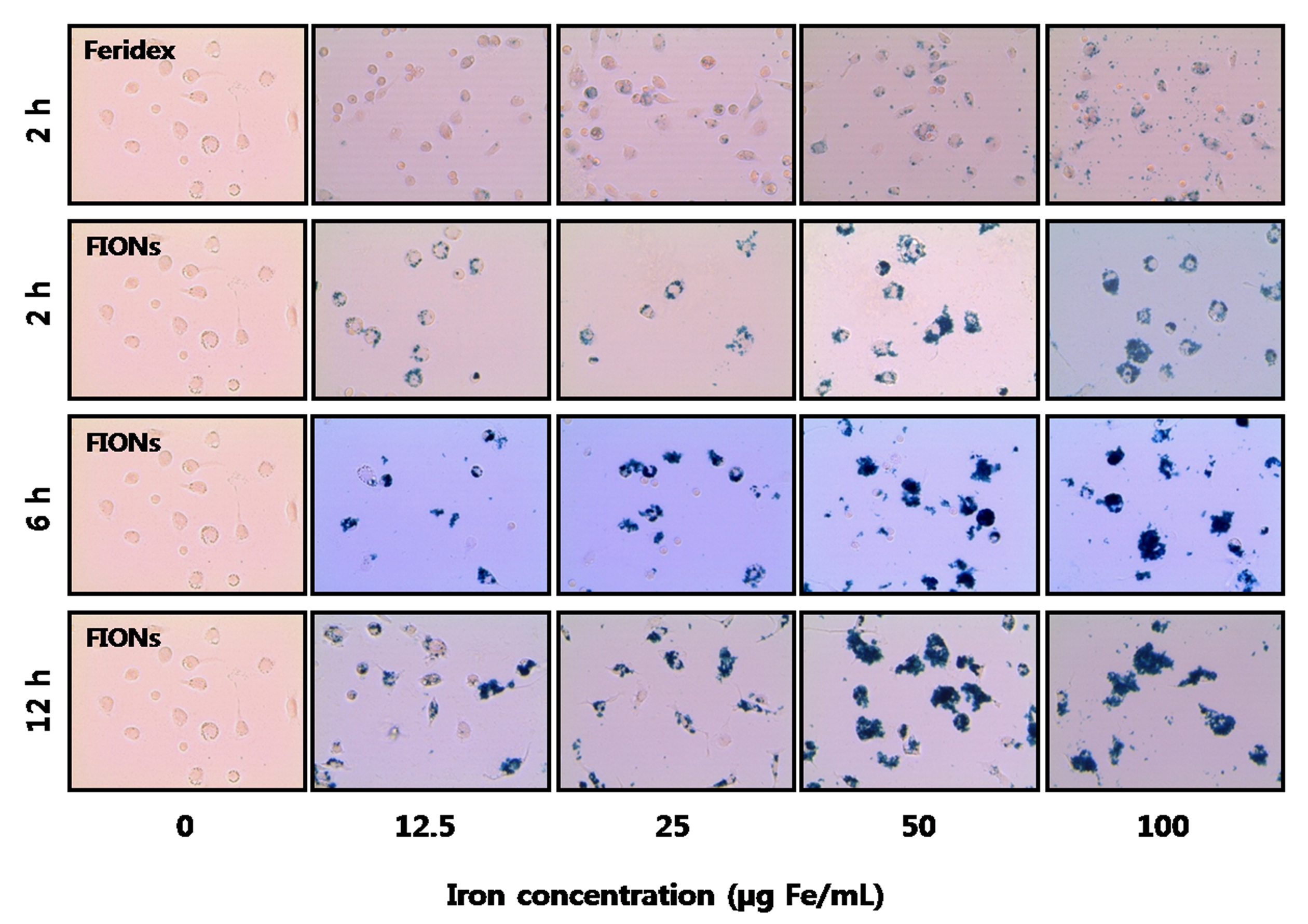

Supplement: Figure S1 — The Prussian blue staining of macrophages after incubation with various concentrations of iron oxide for different times. With increases in the concentration of iron oxide (from 12.5 to 100 µg Fe/mL) and exposure time (from 2 to 12 h), the macrophages phagocytosed more particles. When compared with Feridex-labeling, the FION-labeled macrophages showed a higher intracellular uptake of iron after a 2 h-incubation period. (TIF) [file pone.0029575.s001.tif]

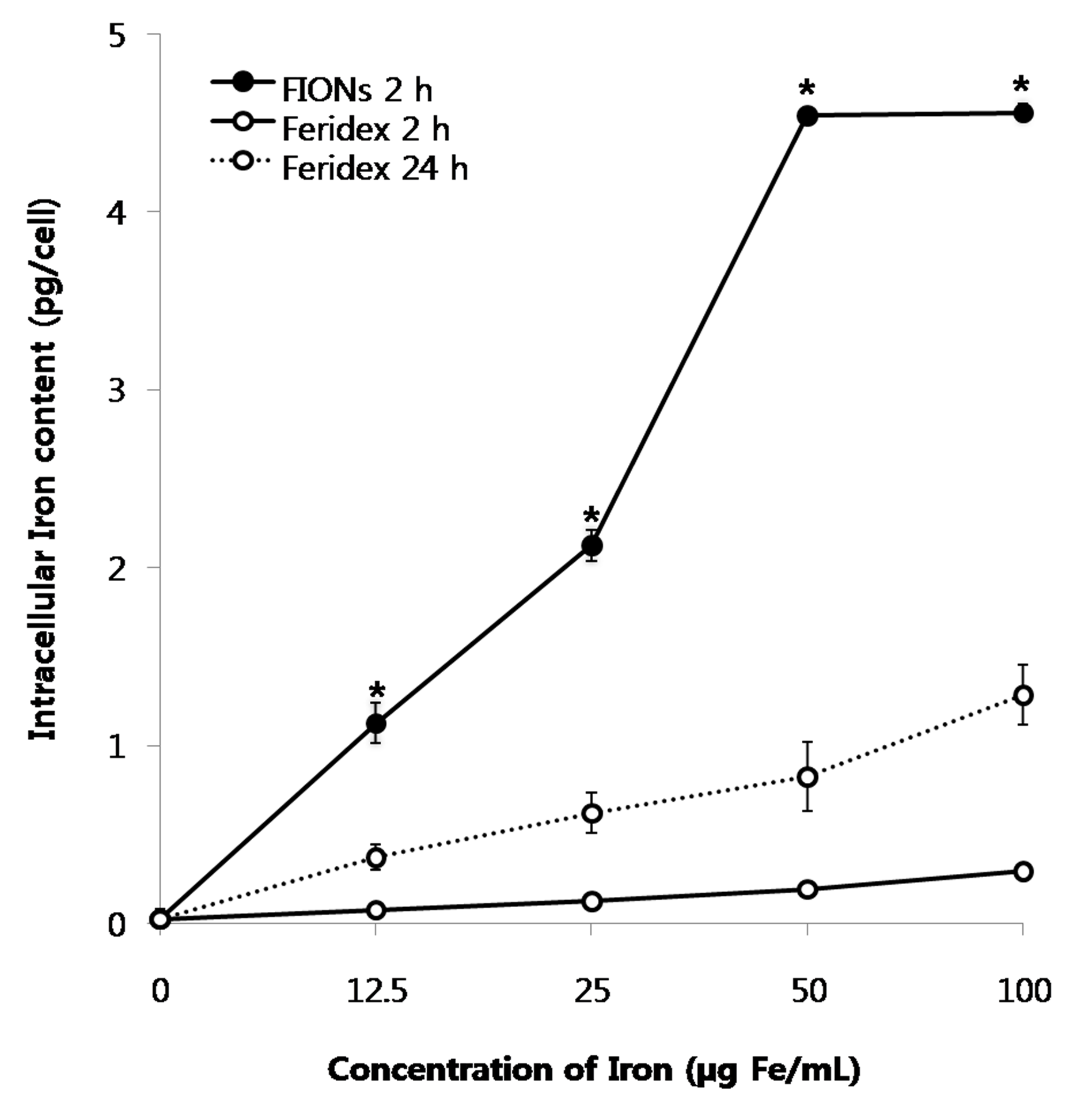

Supplement: Figure S2 — The intracellular iron content (pg Fe/cell) after incubation with increasing iron oxide concentrations (µg Fe/mL). The iron concentration in the macrophages correlated to the FION incubation concentration. Up to 4.539 (pg) of FIONs could be ingested by one macrophage when the FION incubation concentration reached 50 (µg Fe/mL) for 2 h. The amount of iron was 2.214 (pg) when the FIONs concentration was 25 (µg Fe/mL) for 2 h (•). However, when the macrophages were incubated with Feridex in concentration 100 (µg Fe/mL) for 24 h, the amount of iron ingested was 1.284 (pg/cell) (○ with the dotted line), which was significantly lower (* P<0.001) than the iron content of the macrophages treated with different concentration of FIONs (from 12.5–50 µg Fe/mL) for 2 h. (TIF) [file pone.0029575.s002.tif]
