# Supplementary material for: Macrophages Homing to Metastatic Lymph Nodes Can Be Monitored with Ultrasensitive Ferromagnetic Iron-Oxide Nanocubes and a 1.5T Clinical MR Scanner
Source: PLoS One. 2012 Jan 10;7(1):e29575. doi: 10.1371/journal.pone.0029575 (PMC3254614; doi:10.1371/journal.pone.0029575)
Supplement: File S1 — Supporting information. (DOC) [file pone.0029575.s003.doc]

To evaluate the capacity of murine macrophages to phagocytose the MR contrast agent, Prussian blue staining and an assessment of the intracellular iron concentration were performed. For Prussian blue staining, peritoneal macrophages were incubated with FIONs or Feridex (Advanced Magnetics, Cambridge, MA) at concentrations of 12.5, 25, 50, or 100 µg of iron per milliliter for varying times (2, 6, or 12 h) in standard tissue culture incubators with 5% CO_2_. After incubation, cells were washed using PBS and fixed using 4% paraformaldehyde. Cells were then incubated with a 1:1 (vol/vol) mixture of 1% potassium ferrous cyanide and 5% HCl for 30 min. After the incubation, the cells were rinsed in PBS and counterstained with Nuclear Fast Red for 10 min. The presence of iron oxide was qualitatively assessed with a microscopic (Leica). To determine the iron concentration in the macrophages, the macrophages were incubated with FIONs or Feridex at concentrations of 12.5, 25, 50, or 100 µg/mL for 2 h. The macrophages were washed three times with PBS, and 2 x 10^5^ cells were harvested, resuspended in a 6 N solution of HCl, and incubated at 70 ºC for 30 min. The iron amount was determined using a total iron reagent kit (Pointe Scientific, Canton, MI). The average iron load was calculated by dividing the total mean iron content by the cell number.

Increases in the concentration of iron oxide (from 12.5 to 100 µg Fe/mL) and exposure time (from 2 to 12 h) caused, the macrophages to phagocytose more particles, which was demonstrated using Prussian blue staining and optical imaging (Figure S1). When compared with Feridex labeling, the FION-labeled macrophages showed a higher intracellular uptake of iron after a 2 h-incubation period.

**Figure S1.** **The Prussian blue staining of macrophages after incubation with various concentrations of iron oxide for different times.** With increases in the concentration of iron oxide (from 12.5 to 100 µg Fe/mL) and exposure time (from 2 to 12 h), the macrophages phagocytosed more particles. When compared with Feridex-labeling, the FION-labeled macrophages showed a higher intracellular uptake of iron after a 2 h-incubation period.
