# Supplementary material for: Macrophages Homing to Metastatic Lymph Nodes Can Be Monitored with Ultrasensitive Ferromagnetic Iron-Oxide Nanocubes and a 1.5T Clinical MR Scanner
Source: PLoS One. 2012 Jan 10;7(1):e29575. doi: 10.1371/journal.pone.0029575 (PMC3254614; doi:10.1371/journal.pone.0029575)
Supplement: File S2 — Supporting information. (DOC) [file pone.0029575.s004.doc]

The iron concentration in the macrophages correlated to the FION incubation concentration (Figure S2). Up to 4.539 pg of FIONs could be ingested by one macrophage when the incubation concentration reached 50 µg Fe/mL for 2 h. The amount of ingested FION did not increase, significantly when the incubation concentration was increased to 100 µg Fe/mL. When the macrophages were incubated with Feridex (100 µg Fe/mL) for 24 h, the amount of iron ingested was 1.284 pg/cell, which was significantly less (P < 0.001) than the ingested iron content of the macrophages treated FIONs (50 µg Fe/mL) for 2 h. On the basis of these results, the macrophages were labeled with 50 µg Fe/mL FIONs for 2 h for all subsequent experiments.

**Figure S2. The intracellular iron content (pg Fe/cell) after incubation with increasing iron oxide concentrations (µg Fe/mL).** The iron concentration in the macrophages correlated to the FION incubation concentration. Up to 4.539 (pg) of FIONs could be ingested by one macrophage when the FION incubation concentration reached 50 (µg Fe/mL) for 2 h. The amount of iron was 2.214 (pg) when the FIONs concentration was 25 (µg Fe/mL) for 2 h (●). However, when the macrophages were incubated with Feridex in concentration 100 (µg Fe/mL) for 24 h, the amount of iron ingested was 1.284 (pg/cell) (○ with the dotted line), which was significantly lower (* *P* < 0.001) than the iron content of the macrophages treated with different concentration of FIONs (from 12.5 - 50 µg Fe/mL) for 2 h.
